# Optimal diving: patch quality, depth, and marginal value

**DOI:** 10.1093/beheco/araf141

**Published:** 2025-11-29

**Authors:** Alasdair I Houston, Annette Fayet, John M McNamara

**Affiliations:** School of Biological Sciences, University of Bristol, Life Sciences Building, 24 Tyndall Avenue, Bristol BS8 1TQ, United Kingdom; Norwegian Institute for Nature Research (NINA), Høgskoleringen 9, Trondheim 7034, Norway; School of Mathematics, University of Bristol, Fry Building, Woodland Road, Bristol BS8 1UG, United Kingdom

**Keywords:** dive depth, Marginal Value Theorem, oxygen depletion, patch quality

## Abstract

The Marginal Value Theorem, a widely used model of how long an animal should spend foraging on a given patch, has often been invoked in the context of diving animals to predict optimal underwater foraging time. Here, we highlight and address two main issues regarding using the Marginal Value Theorem in this context. First, we show that the theorem’s central assumption of diminishing returns from foraging may not always be correct or necessary, and provide an analysis demonstrating that both ecological and physiological influences on patch residency time—based on prey abundance and aerobic capacity, respectively—which have sometimes been presented as alternatives are, in fact, both important and interacting. Second, we attempt to clarify common confusions around interpreting how environmental quality should affect optimal foraging time, in the cases of homogeneous and heterogenous habitats, for which the effect of quality differ. Finally, we discuss a case in which the foraging gain depends on both foraging time and depth, and prove that the optimal foraging depth is not necessarily the depth at which the energetic rate of gain peaks. Altogether, the clarifications and general proofs we provide should improve future interpretations of models of optimal foraging in diving animals.

## Teaser

How long should a diving animal spend foraging underwater? We give an equation that shows how both the foraging conditions and the animal's physiology determine the optimal behaviour. The time needed to replace oxygen used in the dive can make it optimal to return to the surface even if the rate of intake of food is increasing. We also establish how environmental quality should affect the optimal foraging time.

## Introduction

The Marginal Value Theorem (hereafter MVT) has been an influential contribution to the field of optimal foraging theory. The seminal paper by Charnov (1976) presents a model of the optimal time an animal foraging in a patchy environment should spend feeding in a given patch and is based on the assumption of diminishing returns (ie the rate at which energy is gained decreases perhaps because the patch becomes depleted over time). The model provides a simple criterion for when an animal should leave a patch, which is when the marginal rate is equal to the average rate for the environment (eg Stephens and Krebs 1986). This model has been used in many systems, from birds, insects, fish, and rodents to plants and even humans (Cowie 1977; Giraldeau and Kramer 1982; Pleasants 1989; McNickle and Cahill 2009; Pacheco-Cobos et al. 2019) and has underpinned numerous other models of patch residency time (Brown 1988; Todd and Kacelnik 1993; Calcagno et al. 2014). The MVT has often been invoked in the case of diving animals, which forage underwater but must return to the surface to breathe, such as diving seabirds and pinnipeds. Kramer (1988) talks about using the MVT to analyze the optimal use of oxygen, an approach that has been very influential (Houston and Carbone 1992; Mori 1998a, 1998b; Houston 2011, 2021). To avoid ambiguity, we do not refer to this approach as an example of the MVT. Instead we confine the term to its original context as a specification of optimal foraging time when the rate of energy intake decreases with time in a patch. Empirical papers have used the MVT or ideas from central place foraging Orians and Pearson (1979) to predict how patches of different quality should be exploited (Tome 1988, 1989; Thums et al. 2013; Watanabe et al. 2014; Bestley et al. 2015; Foo et al. 2016; Sutton et al. 2021). However, invoking the MVT in this context can be problematic. One particular issue relates to the MVT’s central assumption of diminishing returns from foraging. This assumption may not always be correct in the diving context, as evidenced by multiple empirical studies measuring a diver’s food intake at a given patch and finding constant or accelerating gains (Watanabe et al. 2014; Ferraro et al. 2017; Sutton et al. 2021), and may also not be necessary for there to be an optimal dive duration Houston and Carbone (1992). We address this issue by establishing some general results about optimal foraging time in diving animals. In doing so, we also argue against previous attempts to present physiological approaches (based on oxygen depletion and recovery) and ecological approaches (based on prey abundance, etc) as competing (Bestley et al. 2015; see also Hazen et al. 2015), by demonstrating how both are important and interact Houston (2021) and proposing an analysis that unifies the two. Another key issue relates to the frequent confusion in empirical papers concerning the interpretation of the MVT, especially with regards to predictions of how changes in environmental quality should affect optimal foraging time. Such predictions are often made incorrectly and without a formal model, and it is common to read opposite interpretations of the MVT, for instance that animals encountering a higher quality patch should forage for longer (Foo et al. 2016; Ferraro et al. 2017; Sutton et al. 2021) or should reduce their foraging (Thums et al. 2013; Bestley et al. 2015). In fact, making such predictions is not straightforward, even in the “standard” MVT case without the additional complication of the aerobic capacity of diving animals. A reason for this may arise from the lack of a clear definition of “quality” (Fayet and Houston 2021), and the fact that different ways of changing quality can affect patch residency time differently (Calcagno et al. 2014; Fayet and Houston 2021). Our aim is to bring ecological and physiological factors together in a single unified model that we use to clarify how changes in environmental quality can have complex effects on optimal foraging time, in particular by exploring the effects of quality in the two cases of homogenous environments (all patches are the same quality) vs heterogenous environments (patches differ in quality), for which predictions of optimal strategies greatly differ Calcagno et al. (2014).

## Model assumptions

Our general approach is to find the foraging behavior that maximizes the rate of energetic gain. In doing so we use what Houston (2011) calls time allocation models. These models are not concerned with the capture of individual items but assume that the energetic gain from foraging is given by the time spent foraging in a particular area but not by the time traveling to and from this foraging area. They also assume that the uptake of oxygen at the surface is subject to diminishing returns. In contrast, the rate of gain from foraging is often assumed to be constant. This approach has been applied to a range of species (eg Tome 1988, 1989; Carbone and Houston 1994; Croll et al. 2001; Mori et al. 2002; Halsey et al. 2003; Doniol-Valcroze et al. 2011; Watanabe et al. 2014; Hazen et al. 2015; Foo et al. 2016; Tyson et al. 2016; Sutton et al. 2021). We use the framework of Houston and Carbone (1992) to establish some general results (eg equation (5) and the conditions for optimal foraging time to increase with quality), and illustrate them with numerical calculations in particular cases. We are not criticising any previous models; instead we use a standard approach to establish their implications.

Under optimal diving theory, animals can optimize two parameters during a dive: the depth at which they dive to forage, and the time they spend foraging. We follow previous models (eg Mori 1998a; Houston and Carbone 1992) in assuming that depth is uniquely described by the time spent traveling to the foraging area and back to the surface. The full dive cycle duration can be described by t+τ+s(t,τ), where *t* is the time spent foraging, *τ* the time spent traveling from and back to the surface, and *s* is the time at the surface before the dive, also called surface-pause duration, which is a function of the time to be spent underwater. *s* depends on the animal’s physiology, while also being affected by environmental conditions such as temperature (Grémillet et al. 2001; Hedd et al. 2009). Initially we fix the dive depth, and hence fix *τ*, investigating the optimal foraging time at this depth.

The environment has different types of foraging patch. The frequency with which the forager encounters a patch of type *q* is $p_{q}$. If the foraging animal spends time *t* foraging on a patch of type *q* its gain is $g_{q}(t)$. This gain function can potentially take multiple forms and have different numbers of parameters (Fayet and Houston 2021). Patch types are encountered at random, but the diver is assumed to know the type before it begins its dive. We return to this assumption in the Discussion. Using this knowledge, the diver decides on the time, *t*, spent foraging on the patch before the dive and stores enough oxygen to end the dive at the surface with no oxygen. The rate of oxygen uptake decreases as oxygen stores increase (Kramer 1988; Houston and Carbone 1992). This means that the time on the surface s(t+τ) is an accelerating function of the foraging time *t*. A dive cycle starts when the forager surfaces from its previous dive and ends when it surfaces again. The gross rate of gain across the environment (*R*) is the expected energy from a cycle (*E*) divided by the expected duration of the cycle (*D*) (Houston and McNamara 1999).

If an animal employs the strategy of spending time $t_{q}$ foraging on type *q* patches, the expected energy is


(1) $$E=\sum_{q}p_{q}g_{q}(t_{q})$$

and the expected duration is


(2) $$D=\tau +\sum_{q}p_{q}[t_{q}+s(t_{q}+\tau )].$$

The gross gain rate under this strategy is thus


(3) $$R=\frac{\sum_{q}p_{q}g_{q}(t_{q})}{\tau +\sum_{q}p_{q}[t_{q}+s(t_{q}+\tau )]}.$$

The optimal gross rate across the environment, $R^{*}$, is the maximum value of *R*.

## Results

### The MVT in a diving context

For each patch type *q* let $t_{q}^{*}$ be the value of *t* that maximizes


(4) $$g_{q}(t)-R^{*}[t+\tau +s(t+\tau )].$$

Then, as demonstrated in McNamara (1982) and Houston and McNamara (1999), the strategy of foraging for time $t_{q}^{*}$ on a patch of type *q* maximizes the long-term rate of gain in the environment. Differentiating expression (4) to find the maximum, we deduce that


(5) $$\frac{g_{q}^{\prime }(t_{q}^{*})}{1+s^{\prime }(t_{q}^{*}+\tau )}=R^{*}\;\text{for each}\;q.$$

Note that $g_{q}^{\prime }(t)$ is the marginal rate of energy gain after time *t* on a patch of type *q*. Motivated by equation (5), we can also define a modified marginal gain rate


(6) $${\tilde{g}}_{q}^{\prime }(t)=\frac{g_{q}^{\prime }(t)}{1+s^{\prime }(t+\tau )}.$$

This modified marginal rate depends on the actual marginal rate, but also on $s^{\prime }$ which is the rate of increase of surface time with increased dive time. Under the MVT a patch of type *q* would be left when $g^{\prime }$ falls to $R^{*}$. In contrast, for a diving animal a patch of type *q* is left when ${\tilde{g}}_{q}^{\prime }$ falls to $R^{*}$. Thus under the MVT the value of $g_{q}^{\prime }$ at which the animal ceases foraging is equalized across patches. In contrast, for a diving animal the value of ${\tilde{g}}_{q}^{\prime }$ is equalized across patches.

If all patches in the environment are equal with gain function g(t), an animal that spends time *t* foraging on each patch has gross gain rate


(7) $$R(t)=\frac{g(t)}{t+\tau +s(t,\tau )}.$$

There is a single optimal dive time $t^{*}$ satisfying ${\tilde{g}}^{\prime }(t^{*})=R^{*}$, where


(8) $${\tilde{g}}^{\prime }(t)=\frac{g^{\prime }(t)}{1+s^{\prime }(t+\tau )}.$$

**Fig. 1.**
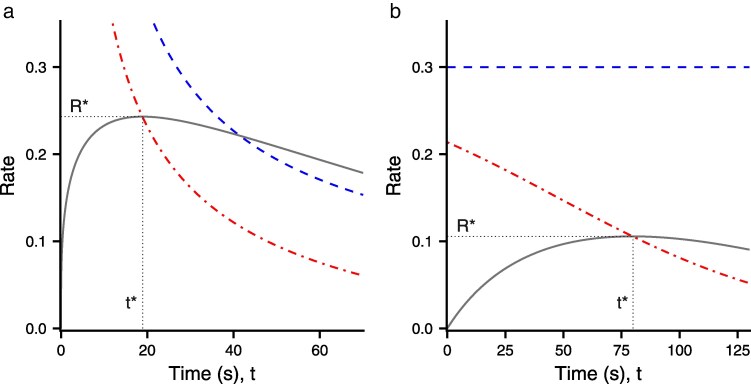
The overall rate R(t) (grey unbroken curve), the marginal rate of energetic gain $g^{\prime }(t)$ (blue dashes) and the modified marginal rate ${\tilde{g}}^{\prime }(t)$ given by equation (8) (red dots) as a function of foraging time *t*. ${\tilde{g}}^{\prime }(t)$ crosses R(t) at the optimal foraging time $t=t^{*}$. The resulting optimal rate is $R^{*}$. In both cases $g(t)=qt^{x}$, s(t,τ)=aexp(c(t+τ)) with a=3.18, c=0.019 (from Elliott et al. 2008) and τ=50. a) q=10,x=0.3, b) q=0.3,x=1.0.

Note ${\tilde{g}}^{\prime }(t)$ is a rate of change of energy with respect to time divided by a rate of change of time with respect to time and hence is a rate with the dimension of energy/time. Diminishing returns in oxygen uptake mean that surface time *s* accelerates with time underwater, so $s^{\prime }$ increases with time. The equation ${\tilde{g}}^{\prime }(t^{*})=R^{*}$ is illustrated in Fig. 1 for the gain function $g(t)=qt^{x}$ used by Mori et al. (2002), where *q* is a constant. The figure plots R(t) and the modified marginal rate ${\tilde{g}}^{\prime }(t)$ as a function of foraging time *t*. It can be seen that ${\tilde{g}}^{\prime }(t)$ drops to R(t) at $R^{*}$. The figure also shows the marginal rate $g^{\prime }(t)$. In Fig. 1a, x<1 so $g^{\prime }(t)$ decreases, but it crosses R(t) at a time that is greater than the optimal foraging time $t^{*}$. In Fig. 1b, x=1 so $g^{\prime }(t)$ is constant and does not cross R(t). Thus the MVT equation cannot hold but there is an optimal foraging time $t^{*}$.

Since expression (4) is maximized at $t=t^{*}$, the second derivative of expression (4) must be negative at $t=t^{*}$ so that


(9) $$g^{\prime \prime }(t^{*})-s^{\prime \prime }(t^{*}+\tau )R^{*}<0.$$

In the standard MVT, $s^{\prime \prime }=0$ for all arguments so there is no effect from *s*, and therefore $g^{\prime \prime }(t^{*})$ must be negative. In a diving context, $g^{\prime \prime }(t^{*})$ can be positive if $s^{\prime \prime }(t^{*}+\tau )$ is large enough. In other words, the penalty in terms of increased surface time imposed by being under water is sufficient to bring the diver back to the surface, even if it is not experiencing diminishing returns from foraging (Houston and Carbone 1992).

### Dive times when patches in an environment differ in quality

When quality differs between patches in an environment (what Calcagno et al. 2014 refer to as a heterogeneous habitat), a key question is whether it is optimal for the animal to spend more time on better patches. We show that the answer depends on circumstances.

We regard the parameter *q* as indicating patch quality. It is convenient to change notation slightly in this section and write $g_{q}(t)$ as g(t,q). For simplicity, we assume that gain g(t,q) on a patch is an increasing function of the time spend foraging *t* and patch quality *q*. We also denote the optimal value of *t* in a patch of quality *q* by $t^{*}(q)$ rather than $t_{q}^{*}$. In our revised terminology, expression (4) becomes


(10) $$g(t,q)-R^{*}[t+\tau +s(t+\tau )],$$

and this function is maximized when $t=t^{*}(q)$. Differentiating expression (10) with respect to *t* and setting the derivative equal to zero at $t=t^{*}(q)$ gives


(11) $$\frac{\partial g}{\partial t}(t^{*}(q),q)-R^{*}(1+s^{\prime }(t^{*}(q)+\tau ))=0.$$

Note that since expression (10) has a maximum at $t=t^{*}(q)$ the second derivative must be negative:


(12) $$\frac{\partial^{2}g}{\partial t^{2}}(t^{*}(q),q)-R^{*}s^{\prime \prime }(t^{*}(q)+\tau ))<0.$$

We differentiate equation (11) with respect to *q* to obtain


(13) $$\frac{dt^{*}}{dq}=\frac{-\frac{\partial^{2}g}{\partial t\partial q}}{\frac{\partial^{2}g}{\partial t^{2}}-R^{*}s^{\prime \prime }}.$$

By inequality (12)


(14) $$\frac{dt^{*}}{dq}(q)>0\;\Leftrightarrow \;\frac{\partial^{2}g}{\partial t\partial q}>0.$$

In the special case where g(t)=qf(t), where q>0 is the quality parameter and *f* is increasing so $f^{\prime }$ is positive, we obtain


(15) $$\frac{\partial^{2}g}{\partial t\partial q}=f^{\prime }$$

and so


(16) $$\frac{dt^{*}}{dq}(q)>0.$$

**Fig. 2.**
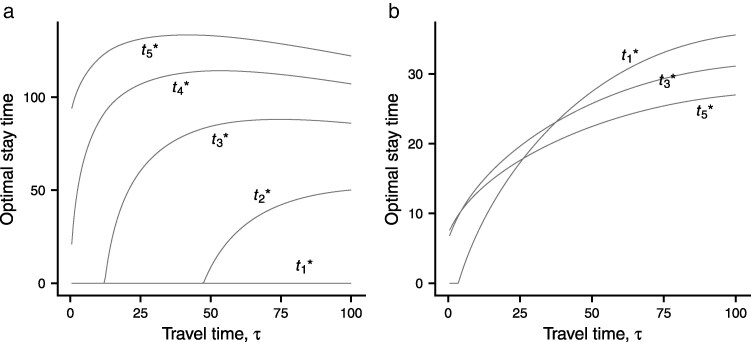
$t^{*}$ as a function of *τ* for a heterogeneous environment with five types of patches, which have with different levels of quality. The five values $q_{1}=0.5,q_{2}=1.0,q_{3}=1.5,q_{4}=2.0,q_{5}=2.5$ are equally likely, so that the probability of each patch type is 0.2. The optimal foraging time on a patch of quality *q* is $t^{*}(q)$. a) Gain function g(t,q)=qt. b) g(t,q)=qt/(20+qt). For clarity of presentation, only three values of $t^{*}(q)$ are shown in this case. s(t,τ)=aexp(c(t+τ)) with a=3.18,c=0.019 (from Elliott et al. 2008).

It follows that the optimal time foraging $t^{*}$ increases with quality. This is illustrated in Fig. 2a for an environment in which quality can take one of five values. As *τ* increases, the environment gets worse ($R^{*}$ decreases) and optimal behavior changes from using the best two patch types, to using the best three, to using the best four. The worst patch type is never used for the range of *τ* illustrated.

For some gain functions, the optimal search time may decrease with increasing quality. Figure 2b illustrates a case in which $t^{*}(q)$ is a decreasing function of *q* for large *τ*.

### Dive times across environments that differ in quality

We now assume that all patches in an environment have the same quality *q* (what Calcagno et al. 2014 call a homogeneous habit), but that *q* can vary across different environments. Our aim is to investigate how foraging times change as *q* changes.

Since all patches in an environment are the same, the foraging times in an environment are the same for each dive. Let R(t,q) denote the rate of gain in an environment of quality *q* if an animal searches for time *t* on each patch. Then


(17) $$R(t,q)=\frac{g(t,q)}{t+\tau +s(t+\tau )}.$$

In the specific case where g(t,q)=qf(t), ie quality multiplies a time-dependent component of the environment, then *q* just rescales the rate and hence $t^{*}(q)$ does not change with *q*. In other words, in this special case, the optimal foraging time in different environments (environments with different *q*) is the same (Houston and Carbone 1992; Mori et al. 2002).

Let z(t,q)=logg(t,q) be the logarithm of the gain function *g*. We introduce the mixed partial derivative


(18) $$M(q)=\frac{\partial^{2}z}{\partial q\partial t}(t^{*}(q),q).$$

The appendix shows that $t^{*}$ increases with quality if and only if


(19) M(q)>0.

We illustrate this condition by comparing


(20) g(t,q)=qt/(20q+t),for whichM(q)>0for allq

and


**Fig. 3.**
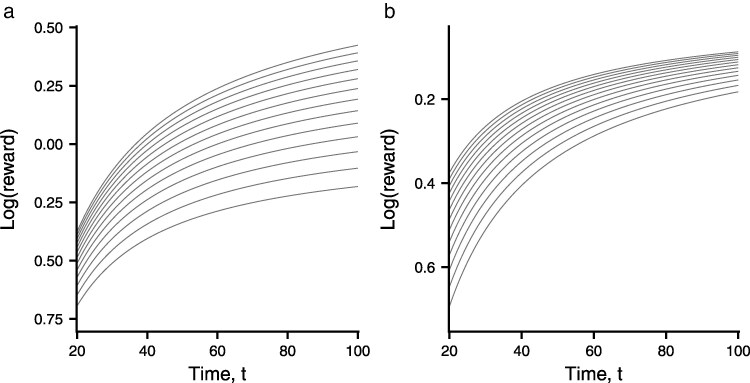
Illustration of z(t,q)=logg(t,q) in two cases. a) g(t,q)=qt/[20q+t]. In this case M(q)>0 so that the lines giving z(t,q) as a function of *t* for constant *q* diverge as *t* increases. b) g(t,q)=qt/[20+qt]. In this case M(q)<0 so that the lines converge as *t* increases.

(21) g(t,q)=qt/(20+qt),for whichM(q)<0for allq.

(These equations are special cases of the function $g(t)=At^{n}/[T^{n}+t^{n}]$ where *A* and *T* are positive constants considered by Fayet and Houston 2021.) Figure 3 contrasts the function z(t,q) for these two cases.

**Fig. 4.**
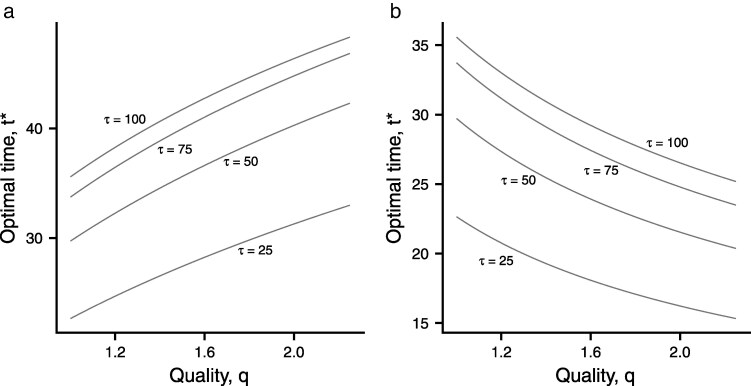
Effect of patch quality *q* on the optimal foraging time $t^{*}(q)$ when all patches in the environment have the same quality. a) g(t,q)=qt/[20q+t], b) g(t,q)=qt/[20+qt].

The rate of increase of z(t,q) with increasing *t* is a measure of the advantage of spending further time on a patch. When M(q)>0 the rate of increase for a given *t* increases with *q*, so that the optimal time $t^{*}(q)$ increases with increasing *q*. In contrast, when M(q)<0 the rate of increase for a given *t* decreases with *q*, so that the optimal time $t^{*}(q)$ decreases with increasing *q*. Figure 4 illustrates $t^{*}(q)$ for the two g(t,q) functions.

### The optimal depth at which to forage

Besides optimizing the foraging time based on the quality of the patch they are feeding on, divers must also decide at which depth to feed. To address this question, we follow the approach of Houston and Carbone (1992) who assume that depth determines the traveling time *τ* and ask what the best value of *t* is. They also assume that gain *g* is described by *bt*, where the rate of energetic gain *b* can depend on depth. At any depth, the best value of *t* maximizes the proportion of time foraging p(t). Mori (1998a) extended the analysis by computing the optimal depth given a particular assumption about the form of the relationship between depth and gain rate *b*, and found that the optimal depth is less than the depth at which *b* is at its maximum. Below we show that this result is true in general. We follow Mori (1998a) in ignoring any effects of depth on metabolism (for which see Wilson et al. 1992) and assuming that the rate of energetic gain from foraging b(τ) depends on depth:


(22) g(t,τ)=b(τ)t.

We assume that b(τ) is a smooth function of food availability with depth. Thus if all patch types are the same, the gross rate of energetic gain becomes


(23) R(t,τ)=b(τ)t/(t+τ+s(t,τ)).

Suppose that this rate is maximized when $t=t^{*}$ and $\tau =\tau^{*}$. Then necessarily the following two conditions hold:


(24) $$\frac{\partial R}{\partial t}(t^{*},\tau^{*})=0$$

(25) $$\frac{\partial R}{\partial \tau }(t^{*},\tau^{*})=0.$$

From equation (24) we obtain


(26) $$t^{*}+\tau^{*}+s(t^{*},\tau^{*})=t^{*}[1+\frac{\partial s}{\partial t}(t^{*},\tau^{*})].$$

This is the standard equation for the maximization of the proportion of time *p* in the dive cycle spent foraging. It follows that


(27) $$p^{*}=\frac{1}{1+\partial s/\partial t(t^{*},\tau^{*})}$$

where $p^{*}=t^{*}/(t^{*}+\tau^{*}+s(t^{*},\tau^{*}))$

From equation (25) it follows that


(28) $$[t^{*}+\tau^{*}+s(t^{*},\tau^{*})]b^{\prime }(\tau^{*})=b(\tau^{*})[1+\frac{\partial s}{\partial \tau }(t^{*},\tau^{*})].$$

This equation determines the optimal depth $\tau^{*}$. Because ∂s/∂τ is positive, it follows from equation (28) that $b^{\prime }(\tau^{*})$ is positive, which means that at the optimal depth, rate of energetic gain *b* is increasing with depth. In contrast, the condition for the travel time (and hence depth) at which *b* is maximized is $b^{\prime }=0$. It follows that if *b* increases and then decreases with depth, the optimal depth is less than the depth at which *b* peaks. Thus the pattern that Mori (1998a) found in his computations is general.

## Discussion

We present a novel general equation for the optimal time spent foraging in a patch which makes it clear how both the gain from foraging, which is related to food availability and hence ecology, and the increase in surface time with time underwater, driven by physiological ability, have an important effect. It can be seen from equation (5) and Fig. 1 that the MVT condition that $g^{\prime }=R^{*}$ does not necessary hold. In fact, the time cost of diving can make it optimal for a diver to return to the surface even when experiencing an increasing rate of gain $g^{\prime }$. Although Houston and Carbone (1992) gave a numerical example that showed this was possible, we have provided a general theoretical explanation. Equation (5) means that diminishing returns from foraging (ie $g^{\prime }<0$) is not necessary for there to be an optimal foraging time. This is illustrated in Fig. 1. Empirical studies found that Adélie penguins (Pygoscelis adeliae) stopped foraging when $g^{\prime }$was increasing in 20% of dives, with 69% of dives classified as showing diminishing returns and 6% as constant Watanabe et al. (2014). Our result means it is possible for there to be an optimal foraging time even when $g^{\prime }$ is increasing.

Understanding how diving behavior is related to patch quality is a fundamental question in foraging ecology, in particular if trying to use diving behavior as an indication of food availability. Calcagno et al. (2014) have shown in the context of the MVT that it is important to distinguish between homogeneous environments, in which all patches are the same, and heterogeneous environments, where patches differ in quality within the environment. We have shown that in the case of diving, this distinction is once again important. We have given conditions on the gain function which determine how optimal foraging time depends on quality.

In heterogeneous environments, where patches can differ in quality, we show that there is an optimal foraging time for a patch given its quality. This is illustrated in Fig. 2, where there are five types of patches in the environment. For the case shown in Fig. 2a, g=qf(t) and at any given travel time *τ*, the optimal foraging time $t^{*}$ increase with quality.

As *τ* increases, $R^{*}$ decreases, therefore the environment becomes worse. When the environment is good, only the best two patches are used, but as $R^{*}$ decreases, there comes a point at which lower quality patches are also used. It can also be seen that for some patch qualities the optimal foraging time first increases and then decreases with *τ*. This pattern is predicted in homogeneous environments (Houston and Carbone 1992; Carbone and Houston 1996; Mori 1999). Viviant et al. (2016) wrongly claim that $t^{*}$ should increase with depth, but find the predicted nonmonotone relationship in Antarctic fur seals (*Arctocephalus gazella*). It is worth noting that *q* has an effect in this case, but does not in homogeneous environments. The effect arises because the parameters of all the types that are used influence $R^{*}$. For the gain function considered in Fig. 2b, the relationship between the optimal foraging time $t^{*}$ and quality depends on depth as characterized by *τ*. When *τ* is large, $t^{*}$ decreases with quality.

In homogeneous environments, we show that the optimal patch time is the same in every patch, and this time increases with quality, provided that


(29) $$M(q)=\frac{\partial^{2}z}{\partial q\partial t}(t^{*}(q),q)>0$$

where z(t,q)=logg(t,q). This condition is explained and illustrated in Figs. 3 and 4. It can be seen from Fig. 4 that $t^{*}$ can either increase or decrease with quality. Which trend is found can be understood in terms of how *t* and *q* determine energetic gain. Mathematically this is represented by the mixed partial derivative M(q), which is indicated by the convergence or divergence of the curves shown in Fig. 3.

Mori (1998b) computed the optimal diving behavior for a particular gain function in heterogenous environments, but it is hard to compare our results with his because in addition to including the possibility of anaerobic respiration, his study maximizes net rather than gross rate and assumes that returns diminish over a series of dives.

To summarize the trends, in homogenous environments, if the gain is given by qf(t) then the parameter *q* has no effect on $t^{*}$. We have also shown (see Fig. 4) that two simple gain functions result in opposite relationships between $t^{*}$ and quality. The situation is different in heterogenous environments. When g=qf(t), increasing the parameter *q* increases $t^{*}$. Once again in the general case $t^{*}$ can increase or decrease with quality, as is shown in Fig. 2. Although the general conditions are complicated, it is noteworthy that they depend on the foraging conditions (as characterized by *g*) but not on the way in which surface time depends on time under water. Given that neither of our conditions depends on physiology (as represented by the function s(t,τ)), it is interesting to see how they compare with the conditions derived by Calcagno et al. (2014). If we ignore the possibility of variation in travel time, then our condition for homogenous habitats is the same as theirs. Our condition is not the same as theirs in heterogeneous habitats, but as far as we can see this is because they consider a wider range of variation than we do.

It is often incorrectly assumed that animals should always forage longer at better patches (Mori et al. 2002), or should always reduce their foraging time (Thums et al. 2013; Bestley et al. 2015). We hope our analysis will help resolve this issue for future studies of diving animals.

Our approach, like that of Mori (1998b), is based on the assumption that the diver knows the quality of the patch that it is about to visit. As a result, it starts the dive with exactly the amount of oxygen that it requires. The next step in modeling optimal diving is to understand the implications of starting a dive without this knowledge.

Although we have mostly focused on the effect of patch quality on optimal diving, we have also considered a case in which the gain from foraging depends on both foraging time and foraging depth. We prove that if the rate of energetic gain *b* increases with depth up to some maximum, and then decreases again, the optimal depth is less than the depth as which *b* is highest. This follows from the fact that at the optimum depth, the rate of increase of gain rate with depth $b^{\prime }$, as given by equation (28), must be positive. We assumed that the gain from a time *t* spent foraging is given by g(t)=b(τ)t, where b(τ) is the rate of energetic gain, which is not easy to measure. If we assume that b(τ)=a(τ)y(τ)e(τ), where a(τ) is food availability, y(τ) is a capture coefficient and e(τ) is the energy content of a prey item, then if *y* and *e* are independent of depth, b(τ)=ka(τ) where *k* is a constant. In this case, we have established analytically that at the optimal depth, food availability as a function of depth is increasing, as found numerically by Mori (1998a) who assumed that b(τ) was normally distributed. Watanabe et al. (2003) found some support for the prediction in the behavior of Weddell seals (*Leptonychotes weddellii*) at one of their study sites while Viviant et al. (2016) found evidence against it in Antarctic fur seals. Neither study involves a conclusive measure of g(t), but Viviant et al. (2016) mentions that energy content of prey or their catchability may change with depth. Foraging behavior has often been used to estimate environmental quality (Mori et al. 2002; Elliott et al. 2008; Shoji et al. 2014; Chimienti et al. 2017). Our analysis of depth shows that an optimal diver may not necessarily chose to forage at the depth where prey is the most abundant, even if it is in reach. This suggests that caution is necessary when using feeding rates as indicators of prey abundance, especially when trying to characterize the environment as a whole.

## Data Availability

No data.

## Appendix

Let g(t,q) be the energetic gain on a patch after time *t* when the environment has quality *q*.

Set

z(t,q)=logg(t,q)

and

y(t)=log[t+τ+s(t+τ)].

Then the gain rate if the diver remains for time *t* in all patches and the environment is *q* is

(A1 )
R(t,q)=exp(z(t,q)−y(t)).

This is maximized over *t* by maximizing z(t,q)−y(t). Denoting the maximizing time by $t^{*}(q)$, we have

(A2 )
$$\frac{\partial z}{\partial t}(t^{*}(q),q)=y^{\prime }(t^{*}(q)).$$

A sufficient condition for a local maximum is

(A3 )
$$\frac{\partial^{2}z}{\partial t^{2}}(t^{*}(q),q)-y^{\prime \prime }(t^{*}(q))<0.$$

Implicitly differentiating with respect to *q* in equation (A2) gives

(A4 )
$$\frac{\partial^{2}z}{\partial t^{2}}(t^{*}(q),q)\cdot t^{*\prime }(q)+\frac{\partial^{2}z}{\partial q\partial t}(t^{*}(q),q)=y^{\prime \prime }(t^{*}(q))\cdot t^{*\prime }(q)$$

so that

(A5 )
$$\frac{dt^{*}}{dq}=\frac{-\frac{\partial^{2}z}{\partial q\;\partial t}(t^{*}(q),q)}{\frac{\partial^{2}z}{\partial t^{2}}(t^{*}(q),q)-y^{\prime \prime }(t^{*}(q))}$$

From this equation and inequality (A3),

$$t^{*\prime }(q)>0\;\text{if and only if}\;\frac{\partial^{2}z}{\partial q\;\partial t}(t^{*}(q),q)>0.$$
